# The role of Cold‐Inducible RNA‐binding protein in respiratory diseases

**DOI:** 10.1111/jcmm.17142

**Published:** 2021-12-24

**Authors:** Peng Zhong, Miao Zhou, Jingjing Zhang, Jianye Peng, Gaofeng Zeng, He Huang

**Affiliations:** ^1^ Department of Cardiology Renmin Hospital of Wuhan University Wuhan Hubei China; ^2^ Cardiovascular Research Institute of Wuhan University Wuhan Hubei China; ^3^ Hubei Key Laboratory of Cardiology Wuhan Hubei China; ^4^ The Second Affiliated Hospital, Department of Cardiovascular Medicine, Hengyang Medical School University of South China Hengyang Hunan China; ^5^ Key Laboratory of Heart Failure Prevention & Treatment of Hengyang Hengyang Hunan China; ^6^ Clinical Medicine Research Center of Arteriosclerotic Disease of Hunan Province Hengyang Hunan China

**Keywords:** acute lung injury, chronic bronchitis, CIRP, pulmonary fibrosis

## Abstract

Cold‐inducible RNA‐binding protein (CIRP) is a stress‐response protein that is expressed in various types of cells and acts as an RNA chaperone, modifying the stability of its targeted mRNA. Intracellular CIRP could also be released into extracellular space and once released, extracellular CIRP (eCIRP) acts as a damage‐associated molecular pattern (DAMP) to induce and amplify inflammation. Recent studies have found that eCIRP could promote acute lung injury (ALI) via activation of macrophages, neutrophils, pneumocytes and lung vascular endothelial cells in context of sepsis, haemorrhagic shock, intestinal ischemia/reperfusion injury and severe acute pancreatitis. In addition, CIRP is also highly expressed in the bronchial epithelial cells and its expression is upregulated in the bronchial epithelial cells of patients with chronic obstructive pulmonary diseases (COPD) and rat models with chronic bronchitis. CIRP is a key contributing factor in the cold‐induced exacerbation of COPD by promoting the expression of inflammatory genes and hypersecretion of airway mucus in the bronchial epithelial cells. Besides, CIRP is also involved in regulating pulmonary fibrosis, as eCIRP could directly activate and induce an inflammatory phenotype in pulmonary fibroblast. This review summarizes the findings of CIRP investigation in respiratory diseases and the underlying molecular mechanisms.

## 1 INTRODUCTION

Cold‐inducible RNA‐binding protein (CIRP) was first discovered two decades ago. It came to the attention of researchers because its expression was induced after cells were exposed to a moderate cold shock.^1^ Later studies showed that the expression of CIRP could also be regulated by hypoxia, UV radiation, glucose deprivation, heat stress and H_2_O_2_, suggesting that CIRP is a general stress‐response protein.^2^ Under stress conditions, CIRP could migrate from the nucleus to the cytoplasm and bind to the 3’‐UTR of target mRNAs. The interaction between CIRP and 3’‐UTRs can influence both the stability and translation of a transcript. A tremendous amount of research on CIRP has revealed its role in the regulation of a variety of cellular stress responses, including cell proliferation, cell survival, the circadian clock, telomerase maintenance, stress adaptation and tumour formation and progression.^3^, ^4^, ^5^, ^6^

Recently, extracellular CIRP (eCIRP) was discovered to be present in various inflammatory conditions and could act as a pro‐inflammatory factor. It has been reported that hypoxia, sepsis and haemorrhagic shock (HS) could induce the release of CIRP into extracellular space.^7^ In vitro work demonstrated that eCIRP can function as a damage‐associated molecular pattern (DAMP) to activate innate Toll‐liker receptor 4 (TLR4)/myeloid differentiation protein 2 (MD2)‐mediated pro‐inflammatory signal to trigger inflammation in macrophages.^8^ In addition, eCIRP was also identified as a new biologically active endogenous ligand of triggering receptor expressed on myeloid cells‐1 (TREM‐1) that incites inflammation in sepsis.^9^ TREM‐1 is also an innate immune receptor expressed primarily on neutrophils and macrophages. Upon activation, TREM‐1 can either directly amplify an inflammatory response or indirectly through synergism with TLR signalling. Targeting CIRP has been demonstrated to be a therapeutic strategy in treating inflammatory diseases. Currently, two CIRP‐derived peptides, which have antagonistic effects on the pro‐inflammatory effects of eCIRP, have been developed. The synthetic oligopeptide, called C23, which is derived from the human CIRP amino acid region (110–125) with the highest affinity to MD2, could block the interaction between CIRP and TLR4/MD2 complex and is reported to exhibit a great potential against CIRP‐induced phagocyte secretion of TNF‐⍺ in macrophages.^8^, ^10^ M3, a 7‐amino acid peptide, which is also derived from the human eCIRP amino acid region (101–107), showed a considerable binding affinity for TREM‐1, could blocks the interaction between TREM‐1 and eCIRP, and exhibited great inhibitory effects on inflammatory cytokine production following eCIRP exposure in macrophages.^9^ These specific peptides with the capacity of blocking the interaction between eCIRP and its receptors could be used as a tool to evaluate the potential role of eCIRP in inflammatory diseases and test the therapeutic potential of targeting CIRP in these conditions.

The lung is an open organ that directly connects to the external environment and is frequently exposed to adverse stimuli, such as cold air, smoke and ambient particulate matter. In addition, the lungs are particularly susceptible to injury in sepsis, and more than 50% of patients with sepsis develop acute lung injury (ALI). Recent studies suggested the involvement of CIRP in the pathogenesis of various types of respiratory diseases, including chronic obstructive pulmonary diseases (COPD), idiopathic pulmonary fibrosis (IPF) and ALI/acute respiratory distress syndrome (ARDS). This article reviews findings on CIRP in respiratory diseases and the underlying molecular mechanism.

## 2 THE ROLE OF CIRP IN COPD AND ITS RESPONSE TO COLD STRESS IN AIRWAY

**FIGURE 1:**
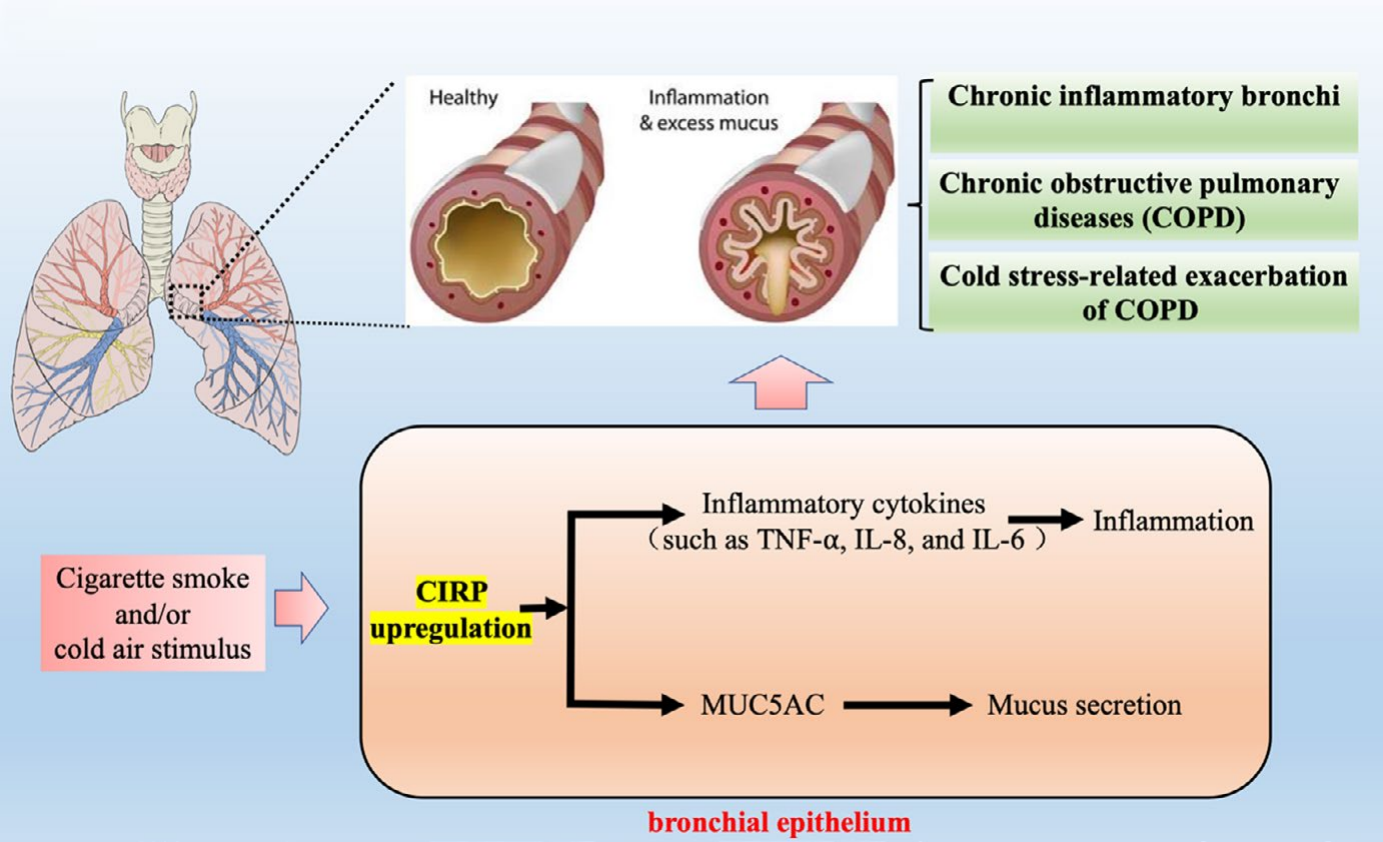
The role of cold‐inducible RNA‐binding protein (CIRP) in bronchial diseases. Under stress conditions, such as cigarette smoke and/or cold air stimulus, the expression of CIRP in bronchial epithelium can be upregulated, which could promote the protein expression of inflammatory cytokines such as TNF‐α, IL‐8 and IL‐6, as well as MUC5AC levels, thus leading to inflammation and excess mucus secretion, contributing to the development of chronic inflammatory bronchi, COPD and the cold stress‐related exacerbation of COPD

Recent studies demonstrated an important role of CIRP in COPD and cold stress‐related exacerbation of COPD (Figure 1). Immunohistochemistry analysis of bronchi samples showed that CIRP protein levels were significantly increased in the bronchial epithelial cells of patients with COPD compared to that of healthy control subjects.^11^ In vivo chronic inflammatory bronchi animal model induced by chronic cigarette smoke exposure, the mRNA and protein expression of CIRP were also increased coupled with upregulated inflammatory cytokines and MUC5AC secretion.^12^ Collectively, these results suggest a possible role of CIRP in the development of COPD.

Cold air stimulus is a major environmental factor that exacerbates chronic inflammatory airway diseases and results in inflammation and mucus hypersecretion.^13^, ^14^ A recent study demonstrated that CIRP was involved in this process. In vivo animal model of chronic inflammatory bronchi diseases induced by chronic cigarette smoke exposure, the expression of CIRP, inflammatory cytokines and MUC5AC secretion could be further exacerbated by cold air stimulation.^12^ Interestingly, CIRP deficiency could significantly inhibit cigarette smoke and cold air‐induced inflammatory response and mucus hypersecretion.^12^ Taken together, these results suggest that CIRP is involved in bronchi inflammation induced by cigarette smoke and contributed to the exacerbated inflammatory response and mucus hypersecretion under cold stress.

The mechanistic study showed that CIRP plays a key role in cold air‐induced mucus overproduction in bronchial epithelial cells. Both in vitro and in vivo studies showed that cold stress (8°C) could induce CIRP expression in bronchial epithelial cells, as well as the protein expression of MUC5AC, which is one of the predominant airway mucins implicated in pulmonary diseases with mucus overproduction.^11^ Interestingly, silencing of CIRP by siRNA in vitro could abolish cold stress‐induced MUC5AC expression in bronchial epithelial cells.^11^ In addition, the expression of TLR4 and phosphorylated NF‐κB p65 (p‐p65) were also increased significantly in response to cold stress in in vitro‐cultured human bronchial epithelial cells. While CIRP siRNA, TLR4‐neutralizing antibody and a specific inhibitor of NF‐κB could attenuate cold stress‐inducible MUC5AC expression.^11^ Moreover, CIRP siRNA could also hinder the expression of TLR4 and p‐p65 induced by cold stress.^11^ Taken together, these results suggest that airway epithelial cells constitutively express CIRP in vitro and in vivo and CIRP is responsible for cold stress‐inducible MUC5AC expression by activating TLR4/NF‐κB signalling pathway.

Similarly, in vitro‐cultured bronchial epithelial cells pretreated with cigarette smoke extract, cold stress could also aggravate the protein expression of CIRP in a time‐dependent manner, coupled with increased protein expression of inflammatory cytokines (TNF‐α, IL‐8 and IL‐6).^12^ However, the mRNA levels of the inflammatory cytokines seem to be unchanged after cold stress, and the protein level of these cytokines is upregulated upon cold stress, suggesting the involvement of post‐transcriptional regulation.^12^ Further studies showed that silencing of CIRP by siRNA could significantly decrease the protein level of the inflammatory cytokines but has no apparent effects on the mRNA level of these cytokines in response to cigarette smoke extract combined with cold stimulus.^12^ These results suggested that CIRP regulates the expression of inflammatory cytokines at the post‐transcriptional level. Mechanistic studies using biotin‐streptavidin pull‐down assays showed that CIRP could bind directly to the 3’‐UTR of inflammatory cytokines such as TNF‐⍺, IL‐8 and IL‐6 mRNA.^12^ CIRP silencing promotes cytokine mRNA degradation and inhibits the inflammatory response.^12^ Collectively, these results suggest that CIRP has no effect on the mRNA expression of cytokines but increased the mRNA stabilization of the inflammatory cytokines to prevent their decay and mediates the expression of inflammatory mediators at the post‐transcriptional level.

Cold‐inducible RNA‐binding protein is a nuclear‐to‐cytoplasmic shuttling protein and can rapidly accumulate in stress granules (SGs) in response to various stress, in which the methylation of arginine residues is necessary for CIRP to be recruited into cytoplasmic SGs.^15^ Interestingly, cold stress was also demonstrated to trigger CIRP translocation and induce the formation of SGs in an arginine methylation‐dependent manner in bronchial epithelial cells. Pharmacological inhibition of methyltransferase could inhibit the translocation of CIRP but did not affect total CIRP expression in response to cigarette smoke extract and cold stress cotreatment.^12^ In addition, the mRNA and protein levels of the cytokines and MUC5AC could be substantially attenuated by the methyltransferase inhibitor in *in vitro* bronchial epithelial cells upon cotreatment stimulation.^12^ CIRP silencing and a methyltransferase inhibitor promote cytokine mRNA degradation and inhibit the inflammatory response. In summary, these results suggest that methylation and cytoplasmic translocation of CIRP is a necessary step for the function of CIRP in bronchial epithelial cells in response to cold stress.

## 3 SEPSIS‐ASSOCIATED ACUTE LUNG INJURY (ALI)

**FIGURE 2:**
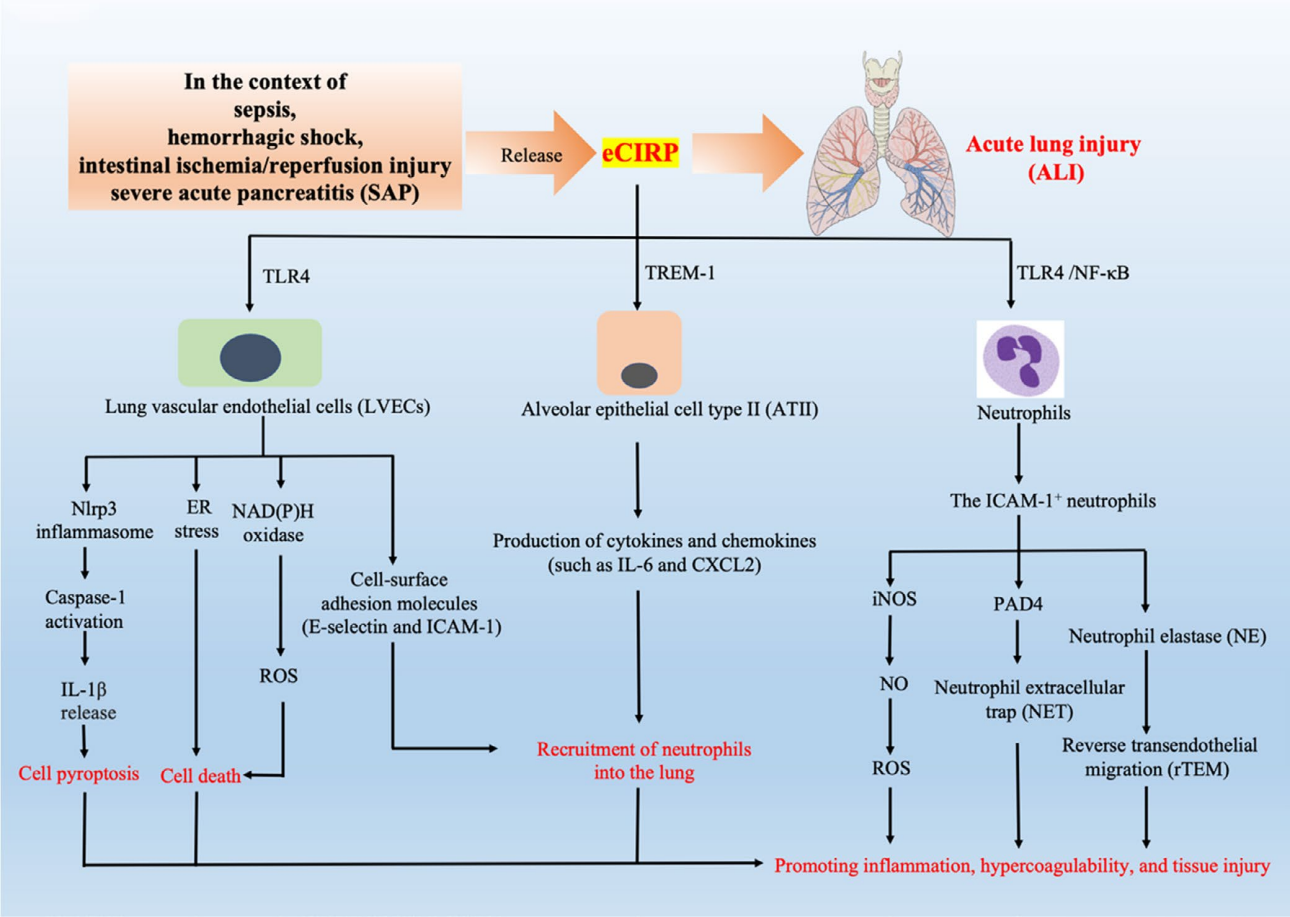
The role of extracellular cold‐inducible RNA‐binding protein (eCIRP) in the development of acute lung injury (ALI). In the context of sepsis, haemorrhagic shock, intestinal ischemia/reperfusion injury and severe acute pancreatitis (SAP), the level of extracellular CIRP (eCIRP) in the circulation can be increased, which can directly induce acute lung injury (ALI) via multiple mechanisms such as activating lung vascular endothelial cells (LVECs), alveolar epithelial cell type II (ATCII) and neutrophils, leading to LVEC cell death, recruitment of neutrophils in the lung, thus promoting inflammation, hypercoagulation and tissue injury

Sepsis is defined as life‐threatening organ dysfunction caused by a dysregulated host response to infection. The lung is particularly susceptible to injury in sepsis, and more than 50% of patients with sepsis develop ALI, clinically defined as acute respiratory distress syndrome (ARDS).^16^ ALI is developed as a consequence of excessive neutrophil infiltration and pulmonary endothelial cell (EC) activation by microbial pathogen‐associated molecular patterns (PAMPs) or endogenous damage‐associated molecular patterns (DAMPs). Recent studies demonstrated a key role of CIRP in mediating sepsis‐associated ALI (Figure 2). Animal studies showed that in the sepsis‐associated ALI model, endoplasmic reticular (ER) stress, inflammatory response, oxidative stress and apoptosis can be observed in lung tissue, while all these changes were abolished in CIRP knockout mice, suggesting an important role of CIRP in the development of ALI during sepsis.^17^ In addition, intravenous injection of recombinant murine CIRP (rmCIRP) in C57BL/6 mice has been shown to cause lung injury, evidenced by vascular leakage, oedema, increased leukocyte infiltration and cytokine production in the lung tissue.^18^ Blockade of CIRP with C23 was also reported to reduce the inflammatory response of the lung in sepsis mice model induced by caecal ligation and puncture (CLP).^10^ C23 treatment was also shown to reduce the mRNA level of IL‐6, IL‐1β, neutrophil chemoattractants, KC and MIP‐2, coupled with a reduction in histological lung injury score in neonatal sepsis mice model.^19^ Injection of a miR‐130b‐3p mimic, which can interact with rmCIRP and decrease the affinity of rmCIRP with its receptor TLR4/MD2, was shown to reduce rmCIRP‐ or CLP‐induced systemic inflammation and ALI in mice.^20^ Collectively, these results demonstrated a critical role of CIRP in the pathogenesis of ALI during sepsis.

Multiple mechanisms have been proposed to contribute to this process. Firstly, during sepsis, intracellular CIRP can be released into the circulation and eCIRP could serve as a DAMP to further induce the release of pro‐inflammatory cytokines from macrophages through TLR4‐dependent manner, amplifying the systemic inflammation.^8^ In addition, eCIRP can also activate CD4^+^ T lymphocytes and predispose CD4^+^ T cells to a Th1 hyperinflammatory response profile and CD8^+^ T cells to a cytotoxic response profile, thus promoting the inflammatory response in sepsis.^21^

Secondly, eCIRP can directly activate lung vascular endothelial cells (LVECs) and induce ER stress and cell death in the lung tissue. A recent study showed that the eCIRP‐induced lung damage mice model is accompanied by lung vascular endothelial cell (LVEC) activation marked by upregulation of cell surface adhesion molecules E‐selectin and ICAM‐1.^18^ Consistently, in vitro‐cultured primary mouse LVEC, rmCIRP treatment could directly increase the ICAM‐1 protein expression and activate NAD(P)H oxidase.^18^ In addition, eCIRP could also stimulate the assembly and activation of Nlrp3 inflammasome in LVEC accompanied with caspase‐1 activation, IL‐1β release and induction of pro‐inflammatory cell pyroptosis.^18^ These results demonstrated that the released CIRP in shock can directly activate LVECs and induce LVEC pyroptosis to cause lung injury. In vivo studies showed that intravenous administration of rmCIRP could result in significant induction of ER stress in the lungs of wild type (WT) mice and immunofluorescence staining of lung tissue showed that ER stress markers were predominantly expressed in pulmonary arteriolar endothelial cells and, to a lesser extent in microvascular endothelial cells, suggesting that eCIRP promotes LVEC activation by inducing ER stress.^17^ Similarly, eCIRP was also shown to induce ER stress in LVECs. Interestingly, administration of rmCIRP to TLR4 knock out (KO) mice failed to induce ER stress in the lung tissue.^17^

Thirdly, CIRP could also cause lung injury by regulating the function of neutrophils. Neutrophils are the most abundant leukocytes in blood and play a pivotal role in host resistance against pathogens. The effector function of neutrophils is mediated through phagocytosis, degranulation, reactive oxygen species (ROS) and neutrophil extracellular traps (NETs).^22^ However, neutrophils are also important mediators of inflammation‐induced injury through the release of cytokines, protease, ROS and NETs.^23^ Although neutrophils are considered a homogenous population of terminally differentiated cells with a well‐defined function, increasing evidence has demonstrated phenotypic heterogeneity and functional versatility of the neutrophil population.^24^ A recent study showed that CIRP could promote the development of the ICAM‐1^+^ neutrophils, which has the properties of producing high levels of ROS during sepsis to cause injury to the lung.^25^ Using a mouse model of CLP‐induced sepsis, ICAM‐1^+^ neutrophils were found to be expanded in the blood and lungs of WT mice, but not in CIRP KO mice after sepsis.^25^ Interestingly, stimulation with rmCIRP could significantly induce ICAM‐1^+^ neutrophils in a time‐ and dose‐dependent manner in vitro bone marrow‐derived neutrophils (BMDN). Interestingly, blockage of TLR4 or NF‐κB with anti‐TLR4 antibody or NF‐κB inhibitor could significantly reduce the frequencies of ICAM‐1^+^ neutrophils in BMDN in response to rmCIRP, suggesting that CIRP‐induced ICAM‐1 expression in neutrophils through the involvement of TLR4‐NF‐κB‐mediated pathway.^25^ In addition, CIRP‐induced ICAM‐1^+^ neutrophils produced much higher levels of iNOS (inducible nitric oxide synthase) and NET compared to ICAM‐1^−^ neutrophils.^25^ As NETs and iNOS were previously shown to be deleterious in sepsis, hence, CIRP‐induced NET/iNOS‐forming ICAM‐1^+^ neutrophils could exaggerate inflammation and injury to the lung during sepsis. Collectively, these results suggest that CIRP plays a key role in regulating iNOS‐producing and NET‐forming ICAM‐1^+^ neutrophils in the lungs during sepsis.

Fourthly, CIRP was also demonstrated to be an inducer of neutrophil extracellular trap (NET) formation in the neutrophils in the lung, which may contribute to lung injury as excessive NET is deleterious to the host via promoting inflammation, hypercoagulability and tissue injury.^26^ A recent study demonstrated that CIRP could induce NET formation in the lung during sepsis and following treatment of healthy mice with rmCIRP.^26^ In vitro‐cultured BMDN, recombinant CIRP was shown to directly induce NET formation via upregulating the expression of PAD4, which has been shown to play a pivotal role in NET formation.^26^ In vivo studies showed that CIRP deficiency could lead to decreased PAD4 expression, coupled with the reduced NET formation in the lung during sepsis.^26^ Taken together, these results suggest that CIRP functions as a novel inducer of NETs by upregulating PAD4 expression in sepsis and demonstrates a novel pathophysiological role of CIRP on promoting ALI during sepsis.

Fifthly, CIRP could regulate the reverse transendothelial migration (rTEM) of neutrophils from lung tissue to the bloodstream during sepsis, which may contribute to the dissemination of systemic inflammation.^22^ Neutrophil migration from the vasculature into the tissue beds is generally regarded as an irreversible and unidirectional mechanism. However, recent studies have reported the ability of neutrophils to return to the bloodstream after migrating to the extravascular space through a process known as rTEM. Neutrophils undergoing rTEM exhibit a pro‐inflammatory phenotype characterized by increased levels of superoxide and high surface ICAM‐1 expression, thus rTEM neutrophils may contribute to turning a local inflammation into a systemic inflammatory response.^27^ The surface phenotypes of reversely migrated (RM) neutrophils are ICAM‐1^high^ CXCR1^low^, whereas the phenotypes of circulating and tissue‐resident neutrophils are ICAM‐1^low^ CXCR1^low^ and ICAM‐1^low^ CXCR1^high^ respectively.^28^ A recent study showed that intratracheal injection of rmCIRP could dose‐dependently increase the frequencies of rTEM neutrophils in the blood.^22^ In addition, the mean frequency of rTEM neutrophils in the blood of CIRP^−/−^ mice was also significantly lower than that of WT mice in the sepsis model.^22^ Collectively, these data demonstrated a major role of CIRP in regulating neutrophil rTEM during sepsis. The frequency of rTEM neutrophils in the blood could be associated with the intensity of inflammation during sepsis, as evidenced by the reduced overall inflammatory response in CIRP^−/−^ mice during sepsis. Further mechanistic study in in vitro‐cultured BMDN showed that rmCIRP could significantly increase neutrophil elastase (NE) levels in the cell supernatants, and when these NE‐rich supernatants were added into endothelial cell (EC) culture, the expression of junctional adhesion molecule (JAM)‐C, which is expressed on the surface of EC and plays a key role in regulating neutrophil rTEM, could be decreased.^22^ This study suggests the direct role of CIRP for inducing neutrophil rTEM by upregulating the expression of NE in neutrophils and subsequently leading to the downregulation of the surface expression of JAM‐C on EC. As these neutrophils acquire inflammatory properties after coming back into the circulation, the rTEM neutrophils may disseminate/re‐enter into other organs to cause multiple organ damage in sepsis. Previous studies have demonstrated the role of CIRP in neutrophil transendothelial migration for infiltration into the lungs to cause ALI after administration of rmCIRP in mice. The identification of such a new role of CIRP in regulating the reverse migration of neutrophils points out a novel pathophysiological role of CIRP in sepsis.

Sixthly, eCIRP could induce the production of cytokines and chemokines in alveolar epithelial cells (AECs), resulting in the recruitment of neutrophils into the lung and causing lung injury.^29^ AECs are an important part of the alveolar‐capillary barrier, which helps with gas exchange and protects the lung from pathogens. A recent study showed that in isolated primary AECs, mostly AEC type II (ATII) cells, treatment with rmCIRP could significantly induce cytokine IL‐6 and chemokine CXCL2 production in a dose‐dependent manner, indicating that eCIRP could induce a pro‐inflammatory phenotype in ATII cells.^29^ As the migration of neutrophils requires the binding of chemokines to chemokine receptors. Interaction between CXCL2 and CXCR2 plays an important role in the recruitment of neutrophils into infection sites. The increased expression of CXCL2 induced by eCIRP in ATII cells could result in a subsequent infiltration of neutrophils into the lung tissue and cause lung injury. A further mechanistic study showed that the pro‐inflammatory effects of eCIRP on ATII cells are mediated through the TREM‐1 pathway. Interestingly, TREM‐1 was also found to be expressed in ATII cells at a low level and TREM‐1 could increase markedly after stimulation with eCIRP. While genetic depletion or pharmacological inhibition of TREM‐1 could decrease the production of IL‐6 and CXCL2 in ATII cells in response to eCIRP or LPS treatment.^29^ Collectively, this study revealed that resting respiratory epithelial cells express TREM‐1 and that secretion of pro‐inflammatory cytokine/chemokine upon exposure to eCIRP is a result of the TREM‐1 signalling pathway.

## 4 HAEMORRHAGIC SHOCK (HS)‐ASSOCIATED ALI

Haemorrhagic shock is associated with an elevated incidence of ALI and ARDS. A recent study showed that CIRP was involved in this process.^30^ C23 peptide which could block the interaction between CIRP and TLR4/MD2 complex was shown to dose‐dependently inhibit TNF‐⍺ release, IκBα degradation and NF‐κB nuclear translocation in macrophages stimulated with eCIRP.^10^ In the HS mice model, lung mRNA levels of IL‐1β, TNF‐α and IL‐6, and lung myeloperoxidase activity were significantly elevated, However, blockage of CIRP by using C23 could significantly reduce the above inflammatory makers in the lung tissues.^30^ M3 was also reported to reduce the systemic and lung inflammatory response in an animal model of haemorrhagic shock (HS), coupled with decreased lung injury score, compared with vehicle‐treated HS mice.^31^ Recently, a new molecular mechanism for mediating the detrimental effects of eCIRP in HS was identified.^32^ In this study, the author demonstrated that HS could cause the release of high levels of eCIRP in the blood, which was then sensed by macrophages via TLR4‐MyD88‐TRIF pathway, leading to the activation of the STING (stimulator of IFN genes) pathway and type 1 IFNs, further exacerbating inflammation.^32^ These results suggest that blockage of eCIRP‐STING signalling could be a potential therapeutic target for mitigating HS‐induced ALI.

## 5 INTESTINAL ISCHEMIA‐REPERFUSION‐INDUCED ARDS

Cold‐inducible RNA‐binding protein was also demonstrated to play a key role in ARDS induced by intestinal ischemia‐reperfusion, as CIRP deficiency could protect the lung in this condition.^33^ Increased levels of pro‐inflammatory cytokines, neutrophil infiltration and apoptosis are the hallmarks of ARDS. After intestinal ischemia‐reperfusion (60 min/20 h), the pro‐inflammatory cytokine IL‐6 protein level, neutrophil infiltration as evidenced by myeloperoxidase (MPO) activity and apoptosis cells in the lung were all elevated significantly, while the CIRP KO mice have significantly decreased levels of the above parameters, coupled with decreased lung injury.^33^ In addition, IL‐6 mRNA levels in the gut tissues and IL‐6 protein levels in the circulation after intestinal ischemia‐reperfusion were also significantly reduced in CIRP KO mice.^33^ These findings strongly established a causal link between CIRP and ARDS during intestinal ischemia‐reperfusion injuries and exposed a possible therapeutic opportunity to mitigate the dreaded complication of ARDS secondary to intestinal ischemia in humans. Recently, blockage of CIRP by intraperitoneal injection of C23 was also reported to decrease neutrophil infiltration and inflammation in the lung as evidenced by reducing expression of IL‐6 mRNA, macrophage inflammatory protein 2 and level of MPO activity in the murine model of intestinal ischemia‐reperfusion.^34^ In addition, treatment with M3 by intraperitoneal injection was reported to reduce local and systemic inflammation in the lung, serum and intestinal in an animal model of intestinal ischemia‐reperfusion injury, coupled with significant resolution of histological injuries of lung and intestinal.^35^ These results indicate that blocking the interaction between eCIRP and or TLR4‐MD2 or TREM‐1 is a promising therapeutic avenue for mitigating intestinal ischemia‐reperfusion injury.

## 6 PANCREATITIS‐ASSOCIATED ALI

Severe acute pancreatitis (SAP) has a high death rate because of its serious complications. SAP can cause multiple organ dysfunction, commonly affecting the lung to induce ALI, even to ARDS, which is responsible for 60%–70% of SAP‐related deaths. Recently, CIRP was demonstrated to be a key mediator in an animal model of SAP‐associated ALI.^36^ In the rat SAP‐ALI model, CIRP protein level in the serum, pancreatic islets and lungs were found to be elevated significantly, coupled with activated NLRP3 inflammasome, increased CXCL1 expression and neutrophil infiltration in the lung of rats.^36^ While treatment with C23 could effectively alleviate the severity of SAP‐induced ALI in rats, as evidenced by improved histological scores of the pancreas and lung sections, decreased serum amylase and IL‐1β levels and elevated PaO2, compared with that of the SAP group.^36^ In vitro‐cultured rat alveolar macrophages, rmCIRP treatment could induce the activation of NF‐κB signalling, inflammasome formation and pyroptosis, which can be significantly mitigated by C23 or TAK242.^36^ Collectively, these results suggested that CIRP is a key inflammatory mediator, contributing to the pathogenesis of SAP‐induced ALI by promoting neutrophil infiltration in the lung.

## 7 CIRP AS A BIOMARKER FOR PREDICTING LUNG DYSFUNCTION IN PATIENTS UNDERGOING CARDIOPULMONARY BYPASS (CPB) SURGERY

During cardiovascular surgery, CPB time is associated with organ dysfunction. Prolonged CPB time induced by complicated cardiac diseases could result in exaggerated inflammatory responses and harmful mediators which could cause multiple organ dysfunction. The lung is among the most vulnerable organs during the perioperative period of cardiovascular surgery with CPB, and 20% of patients who received cardiovascular surgery had ALI during the perioperative period, with a mortality as high as 80%. Recently, the level of plasma CIRP was demonstrated to be a biomarker for predicting lung dysfunction in patients receiving cardiovascular surgery necessitating CPB.^37^ In this study, plasma was collected at different time points during the perioperative period of the cardiovascular surgery with CPB and was then detected for the level of CIRP and inflammatory cytokines, coupled with the collection of other laboratory and clinical parameters at the time.^37^ This study found that plasma CIRP was significantly upregulated immediately after CPB. The elevated levels of CIRP were correlated with the levels of inflammatory cytokines IL‐6 and IL‐10.^37^ Furthermore, the length of CPB time was associated with the level of CIRP production, while CIRP level was correlated with the severity of lung dysfunction.^37^ In brief, these results suggest that plasma CIRP could serve as a predictor for lung injury after cardiovascular surgery with CPB.

## 8 THE ROLE OF CIRP IN IDIOPATHIC PULMONARY FIBROSIS (IPF)

A recent clinical association study suggested that the serum level of CIRP could be used as a biomarker for predicting the outcomes of patients with IPF.^38^ In this study, the author evaluated the association of serum CIRP level upon IPF diagnosis with the disease progression within 1 year after diagnosis. The study showed that the levels of CIRP in serum and lung tissues were higher in patients with IPF than that in control subjects. In addition, the CIRP^high^ subgroup had a significantly higher 1‐year disease progression rate and lower cumulative survival rates than the CIRP^low^ subgroup. More importantly, these results can be replicated in another validation cohort in which the patients were from a different hospital.^38^ Multivariate analysis showed that a high serum CIRP level was independently associated with the higher 1‐year diseases progression and all‐cause mortality rates in both cohorts.^38^ In short, these studies demonstrated that serum CIRP level is a promising biomarker that can help identify high‐risk patients with IPF, especially in the early stage.

**FIGURE 3:**
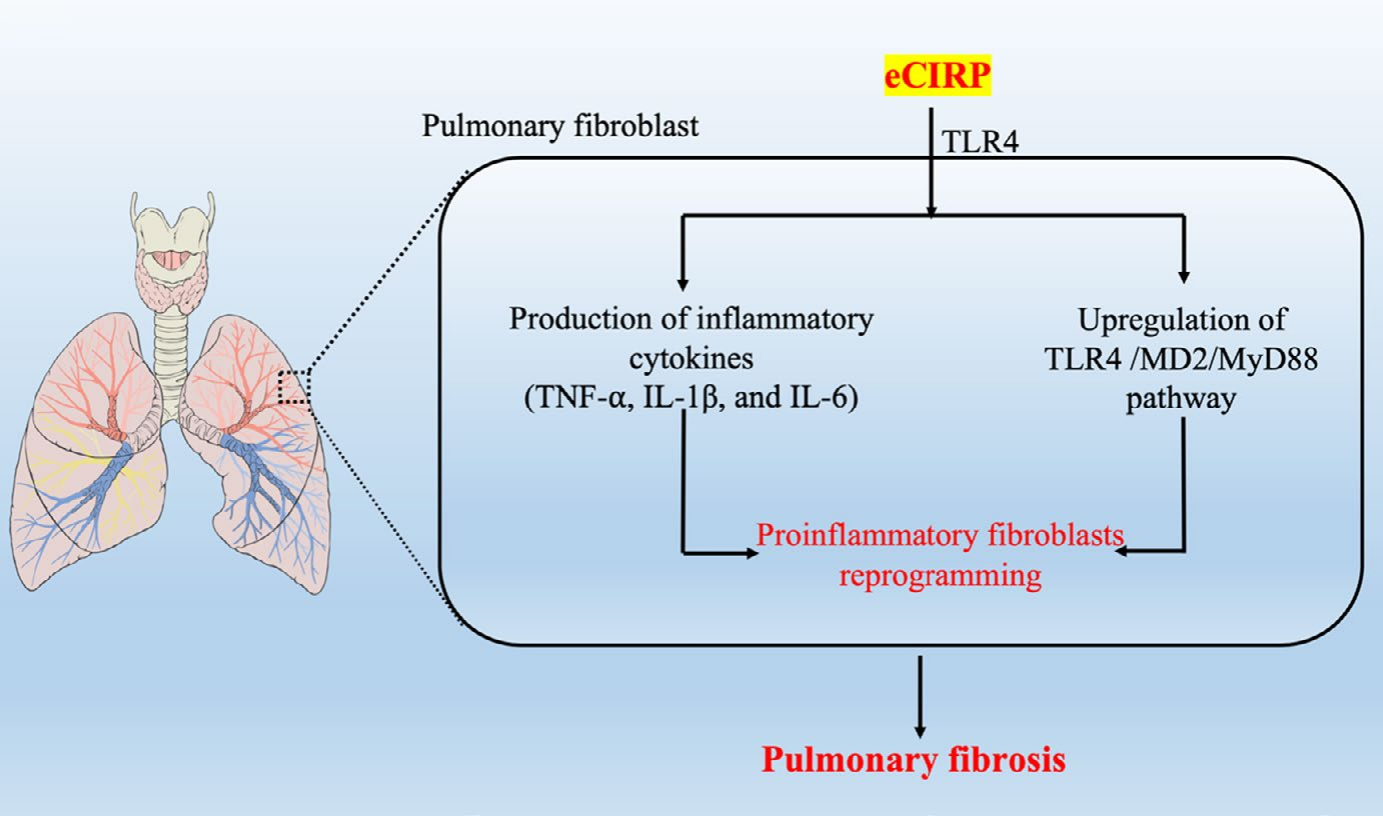
The role of extracellular cold‐inducible RNA‐binding protein (eCIRP) in the development of pulmonary fibrosis. Extracellular CIRP (eCIRP) could directly activate pulmonary fibroblast through the TLR4‐dependent pathway and lead to the production of inflammatory cytokines (TNF‐α, IL‐1β and IL‐6) and the upregulation of TLR4/MD2/MyD88 pathway, leading to the pro‐inflammatory fibroblasts reprogramming, which is a critical step in the development of pulmonary fibrosis

Fibroblasts are the key cells in the development of lung fibrosis. A recent study found that eCIRP could directly induce an inflammatory phenotype in pulmonary fibroblasts via the TLR4 signalling pathway, contributing to pulmonary fibrosis.^39^ It was shown that in vitro treatment with rmCIRP could induce pro‐inflammatory cytokines (TNF‐⍺, IL‐1β and IL‐6) in pulmonary fibroblast in a TLR4‐dependent manner, as all these induced cytokines were almost aborted in TLR4^−/−^ cells.^39^ In addition, treatment with rmCIRP could also increase the expression of the TLR4 coreceptor MD2 and the downstream Myd88 pathway in pulmonary fibroblast in TLR4‐dependent manner.^39^ TGF‐β1 is required for fibrotic activation of the fibroblasts. However, this activation and induction of organ fibrosis require a primed cellular microenvironment, which can be induced by TLR4 activation, suggesting that TLR4‐dependent fibroblast activation is a key driver of persistent organ fibrosis.^40^, ^41^ In the present study, the authors also compared the pro‐inflammatory effects of rmCIRP and rmTGF‐β1 in in vitro‐cultured pulmonary fibroblasts. Principal component analysis of mRNA profile sequenced from WT pulmonary fibroblasts treated with PBS, rmCIRP, rmTGF‐β1 and the combination of the rmCIRP and rmTGF‐β1, showed that eCIRP induces and enriches the pro‐inflammatory phenotype of pulmonary fibroblasts independent of TGF‐β1.^39^ In the animal model of bleomycin‐induced pulmonary fibrosis, there exists an inflammatory‐to‐fibrotic transition phase after bleomycin injection, in which an increase in the lung infiltration by total white blood cells, neutrophils and macrophages peaks on day 14, followed by the expression of profibrotic markers peak on days 21–28. Interestingly, transcriptome analysis of the lung tissues on day 14 after bleomycin injection, showed that the pro‐inflammatory cytokines, TLR4, MD2 and MyD88, and related pathways were all significantly induced, which recapitulate those changes in pulmonary fibroblasts stimulated with eCIRP in vitro.^39^ Collectively, these results suggested a critical role of eCIRP for the pro‐inflammatory fibroblasts reprogramming and may represent a druggable target to stop the progression of pulmonary fibrosis in chronic inflammatory diseases affecting the lung (Figure 3).

## 9 IN SUMMARY AND FUTURE PERSPECTIVE

Despite the extensive studies of ALI/ARDS, there are still no specific pharmacological treatments. To date, the best practice against ALI/ARDS remains protective ventilation, including low tidal volumes and high positive end‐expiratory pressures (PEEP) levels. Nevertheless, the mortality remains unchanged at approximately 40%.^42^ Prone position ventilation, neuromuscular blockade or extracorporeal membrane oxygenation can be used in the short term to manage patients with severe hypoxemia. Also adjuvant therapies such as exogenous surfactants, inhaled nitric oxide may improve arterial oxygenation. However, none of these supportive therapies can reduce inflammation in the lungs nor have been efficiently demonstrated to move forward the overall clinical outcomes of ALI/ARDS. Steroid anti‐inflammatory drugs were supposed to reduce the associated pulmonary inflammation, although their efficacy in ALI/ARDS is debated and the clinical outcomes are controversial.^42^ Accordingly, ALI/ARDS represents an unmet medical need that requires innovative therapeutic strategies to decrease mortalities by treating the underlying molecular mechanisms besides the supportive treatment. The identified critical role of CIRP in promoting the pathogenesis of ALI/ARDS provides us a new therapeutic target in treating this condition. The developed blocker of eCIRP such as C23 and M3 may promote clinical translation of these new therapeutic strategies. The identified role of CIRP in the production of mucus and inflammatory cytokines in bronchial epithelial cells contributes to the development of inflammation and mucus hypersecretion in chronic inflammatory airway diseases and the exacerbation of airway conditions in response to environmental stress. Therefore, targeting CIRP maybe also a therapeutic target in chronic inflammatory airway diseases. The direct pro‐inflammatory effects of eCIRP on pulmonary fibroblast and the predicting role of serum level of CIRP for the progression of idiopathic pulmonary fibrosis in humans suggest a potential role of eCIRP in mediating the development of pulmonary interstitial fibrosis and targeting eCIRP may also have a therapeutic potential in treating pulmonary fibrosis.

## CONFLICT OF INTEREST

The authors declare no conflict of interest.

## AUTHOR CONTRIBUTIONS

**Peng Zhong:** Conceptualization (equal); Funding acquisition (equal). **Miao Zhou:** Investigation (equal). **Jingjing Zhang:** Investigation (supporting). **Jianye Peng:** Conceptualization (equal). **Gaofeng Zeng:** Conceptualization (equal). **He Huang:** Conceptualization (equal).
